# Towards a mathematical theory of meaningful communication

**DOI:** 10.1038/srep04587

**Published:** 2014-04-04

**Authors:** Bernat Corominas-Murtra, Jordi Fortuny, Ricard V. Solé

**Affiliations:** 1 Section for Science of Complex Systems, Medical University of Vienna, Spitalgasse, 23 1090 Vienna, Austria; 2 ICREA-Complex Systems Lab, Universitat Pompeu Fabra, Dr Aiguadé, 88, 08003 Barcelona, Spain; 3 Institut de Biologia Evolutiva. CSIC-UPF. CMIMA-Pssg. de la Barceloneta 37-49. 08003 Barcelona, Spain; 4 Departament de Filologia Catalana. Universitat de Barcelona. Gran Via de les Corts Catalanes, 585, 08007 Barcelona, Spain; 5 Santa Fe Institute, 1399 Hyde Park Road, Santa Fe, New Mexico 87501 USA

## Abstract

Meaning has been left outside most theoretical approaches to information in biology. Functional responses based on an appropriate interpretation of signals have been replaced by a probabilistic description of correlations between emitted and received symbols. This assumption leads to potential paradoxes, such as the presence of a maximum information associated to a channel that creates completely wrong interpretations of the signals. Game-theoretic models of language evolution and other studies considering embodied communicating agents show that the correct (meaningful) match resulting from agent-agent exchanges is always achieved and natural systems obviously solve the problem correctly. Inspired by the concept of *duality of the communicative sign* stated by the swiss linguist Ferdinand de Saussure, here we present a complete description of the minimal system necessary to measure the amount of information that is consistently decoded. Several consequences of our developments are investigated, such as the uselessness of a certain amount of information properly transmitted for communication among autonomous agents.

Major innovations in evolution have been associated with novelties in the ways information is coded, modified and stored by biological structures on multiple scales^1^. Some of the major transitions involved the emergence of complex forms of communication, being human language the most prominent and difficult to explain^2^. The importance of information in biology has been implicitly recognized since the early developments of molecular biology, which took place simultaneously with the rise of computer science and information theory. Not surprisingly, many key concepts such as coding, decoding, transcription or translation were soon incorporated as part of the lexicon of molecular biology^3^.

Communication among individual cells promoted multicellularity, which required the invention and diversification of molecular signals and their potential interpretations. Beyond genetics, novel forms of non-genetic information propagation emerged. At a later stage, the rise of neural systems opened a novel scenario to interact and communicate with full richness^2^. Human language stands as the most complex communication system and, since communication deals with the creation, reception and processing of information, understanding communication in information theoretic terms has become a major thread in our approach to the evolution of language.

In its classical form, information theory (IT) was formulated as a way of defining how signals are sent and received through a given channel with no attention to their meaning. However, in all kinds of living systems, from cells sharing information about their external medium, individuals of a given species surviving in a world full of predators or when two humans or apes exchange signals, a crucial component beyond information is its meaningful content^4^. The distinction is very important, since information has been treated by theoreticians since Shannon's seminal work^5^ as a class of statistical object that measures correlations among sets of symbols, whereas meaning is inevitably tied to some sort of functional response with consequences for the fitness of the communicating agents. This standard scheme describing information transmission through a noisy channel^5^ is summarized in figure (1)a. The most familiar scenario would be described by a speaker (S) and a listener or receiver (R) having a conversation in a living room. The air carries the voice of the first and is the channel, which would be reliable (low or zero noise) if nothing except R and S were present. Instead, the channel will become more and more unreliable (noisy) as different sources of perturbation interfere. These can be very diverse, from air turbulence and children laughing to another conversation among different people. Consistently with any standard engineering design, Shannon's picture allows us to define efficient communication in terms somewhat similar to those used -for example- within electric transmission networks. In this case, a goal of the system design is minimizing the heat loss during the transmission process. Information is a (physically) less obvious quantity, but the approach taken by standard IT is quite the same.

As a consequence of its statistical formulation, IT does not take into account “meaning” or “purpose” which, as noted by Peter Schuster^1^, are also difficult notions for evolutionary biology. Despite this limitation, it has been shown to successfully work in the analysis of correlations in biology^6^. However, one undesirable consequence of this approach is that some paradoxical situations can emerge that contradict our practical intuition. An example is that a given pair of signals *s*_1_, *s*_2_ associated to two given objects or events from the external world could be “interpreted” by the receiver of the messages in a completely wrong way –“fire” and “water”, for example, could be understood, as “water” and “fire”, respectively. Measured from standard IT -see below- the information exchanged is optimal -even perfect- if “fire” (“water”) is always interpreted as “water” (“fire”). In other words, full miscommunication can also score high, as perfectly “efficient”, within Shannon's framework. Therefore, one should approach the communicative sign as a *dual* entity that must be preserved as a whole in the communicative exchange. This crucial *duality sign* in communicative exchanges was already pointed out -with some conceptual differences to the version we will develop below-before the birth of information theory by the Swiss linguist Ferdinand de Saussure in his acclaimed *Cours de linguistique générale*^7^.

It seems obvious that meaning -and its connection to some signal, in order to create the dual entity- plays an essential role and has been shaped through evolution: “the message, the machinery processing the message and the context in which the message is evaluated are generated simultaneously in a process of coevolution”^1^. In our bodies, proper recognition of invaders is essential to survival, and failures to recognizing the self and the non-self are at the core of many immune diseases^8^,^9^. Similarly, learning processes associated to proper identification of predators and how to differentiate them from inmates are tied to meaningful information. Beyond the specific details associated to each system, correct information storing and sharing, and the relevance of meaning is well illustrated by its impact on evolutionary dynamics. As pointed out in^3^ we can say that, in biology, the coder is natural selection. In this way, the use of evolutionary game theoretic arguments has played a very important role in shaping evolutionary approaches to language and commmunication^10^,^11^,^12^,^13^,^14^,^15^, but require some extension in order to properly account for meaningful information. Moreover, evolutionary robotics and the artificial evolution of protolanguages and proto-grammars is a unique scenario where such a framework naturally fits^16^,^17^,^18^,^19^,^20^,^21^,^22^. Evolving robots capable of developing simple communication skills are able of acquiring a repertoire of appropriate signals, share them and interpret correctly the signals sent by other agents. The coherent development of a shared set of symbols that is correctly used -and thus where “meaning” is preserved- becomes central. Such coherence results from the combination of a shared repertoire of signals together with a shared perception of the external world, as detected and perceived by the same class of sensing devices.

In this paper we develop and describe an information-theoretic minimal system in which the signal is linked to a referential value. This relation is assumed to be simple and direct, so that no other process than the mapping is assumed. Other forms of more complex meaning associations would deviate from the spirit of the paper, which is to introduce the minimum framework accounting for the conservation the simplest form of meaning. In a nutshell, we are going to derive an information-theoretic measure able to grasp the consistency of the shared information between agents, when *meaning* is introduced as a primitive referential value attributed to one or more signals.

## Results

We start this section describing the minimal system incorporating referential values for the sent signals. Within this system, we show what is meant when we say that information theory is blind to any meaning of the message. We then derive the amount of consistently decoded information between two given agents exchanging information of their shared world, thereby fixing the problem pointed out above, and analyze some of its most salient properties, including the complete description of the binary symmetric channel within this new framework.

### The minimal system encompassing referentiality

**Figure 2:**
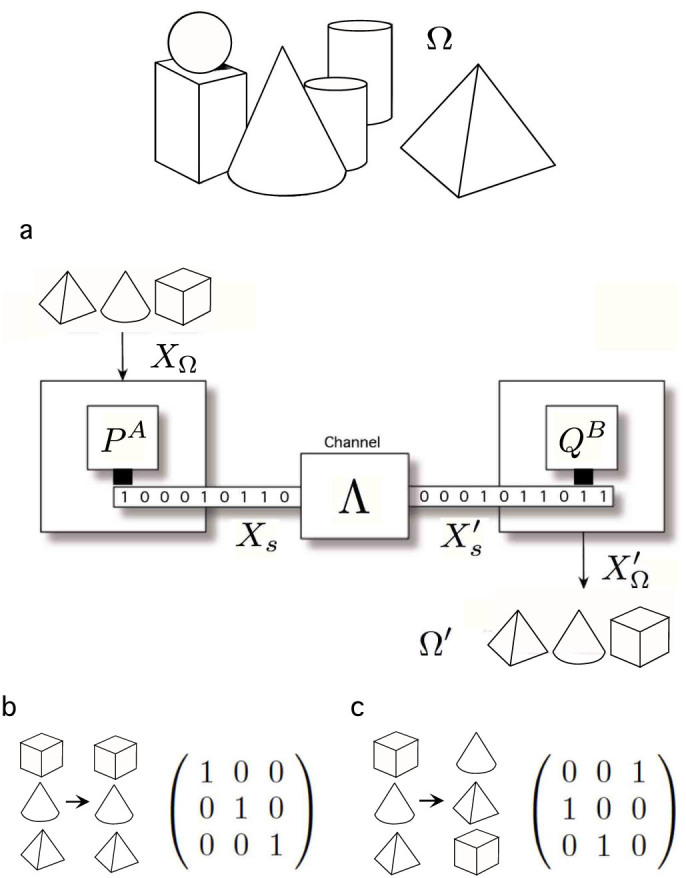
Minimal communicative system to study the conservation of referentiality (a): A shared world, whose events are the members of the set Ω and whose behavior is governed by the random variable *X*_Ω_. A coding engine, **P^A^**, which performs a mapping between Ω and the set of signals 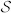, being *X_s_* the random variable describing the behavior of the set of signals obtained after coding. The channel, Λ, may be noisy and, thus, the input of the decoding device, **Q^B^**, depicted by 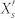, might be different from *X_s_*. **Q^B^** performs a mapping between 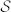 and Ω, whose output is described by 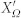. Whereas mutual information provides a measure of the relevance of the correlations between *X*_Ω_ and 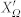, *consistent information* evaluates the relevance of the information provided by consistent pairs with regard to the overall amount of information. In this context, from a classical information-theoretical point of view, situations like b) and c) could be indistinguishable. By defining the so-called consistent information we can properly differentiate b) and c) by evaluating the degree of consistency of input/output pairs -see text.

Our minimal system to study the referential or semantic consistency of a given information exchange will involve two *autonomous communicative agents*, **A**, **B**, a *channel*, Λ, and a *shared world*, Ω. Agents exchange information about their shared world through the channel -see figure (2). Now we proceed to describe it in detail.

#### Description

An *agent*, **A**, is defined as a pair of computing devices, 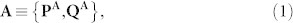
where **P^A^** is the coder module and **Q^A^** is the decoder module. The shared world is defined by a random variable *X*_Ω_, which takes values from the set of events, Ω, Ω = {*m*_1_, …, *m_n_*}, denoting the (always non-zero) probability associated to any event *m_k_* ∈ Ω as *p*(*m_k_*). The coder module, **P^A^**, is described by a mapping from Ω to the set of signals: 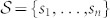. We will here assume 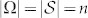, unless the contrary is indicated. The mapping that represents the coder module is defined by means of a matrix of conditional probabilities **P^A^**, whose elements 
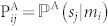
 satisfy the normalization conditions (namely, for all *m_i_* ∈ Ω, 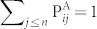). The outcome of the coding process is depicted by the random variable *X_s_*, taking values from 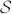 according to a probability distribution 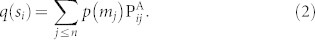
The channel Λ is characterized by the *n* × *n* matrix of conditional probabilities Λ, with matrix elements 
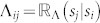
). The random variable 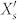 describes the output of the composite system world + coder + channel, thereby taking values on the set 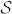, and follows the probability distribution *q*′, defined as 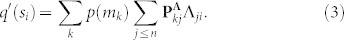
Finally, the decoder module is a computational device described by a mapping from 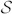 to Ω; i.e. it receives 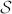 as the input set, emitted by another agent through the channel, and yields as output elements of the set Ω. **Q^A^** is completely defined by its transition probabilities, namely, 
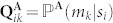
, which satisfy the normalization conditions (i.e., for all 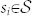, 
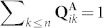
). We emphasize the assumption that, in a given agent **A**, following [14, 15] (but not [10, 11]) there is a priori no correlation between **P^A^** and **Q^A^**.

Now suppose that we want to study the information transfer between two agents sharing the world. Let us consider **A** the encoder agent and **B** the decoder one, although we emphasize that both agents can perform both tasks. Agent **B** tries to reconstruct *X*_Ω_ from the information received from **A**. The description of Ω made by agent **B** is depicted by the random variable 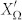, taking values on the set Ω and following the probability distribution *p*′, which takes the form: 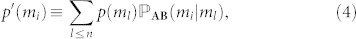
where 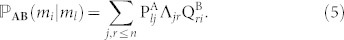
From which we can naturally derive the joint probabilities, 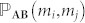 as follows: 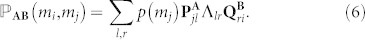
We say that 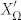 is the *reconstruction* of the shared world, *X*_Ω_, made by agent **B** from the collection of messages sent by **A**. Summarizing, we thus have a composite system where the behavior at every step is described by a random variable, from the description of the world, *X*_Ω_ to its reconstruction, 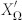 -see figure (2a): 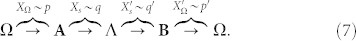
At this point, it is convenient to introduce, for the sake of clarity, some new notation. We will define two matrices, namely **J**(**AB**) and Λ(**AB**) in such a way that 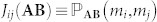 and 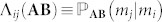. Finally, we will define the probability distribution Λ*_i_*(**AB**) ≡ {Λ*_i_*_1_(**AB**), …, Λ*_in_*(**AB**)}. This new notation will enable us to manage formulas in a more compact way.

#### Information-theorethic aspects of this minimal system

First we shall explore the behaviour of mutual information in this system. Detailed definitions of information-theory functionals used in this subsection are provided in the Methods section. Under the above described framework, we have two relevant random variables: *the world X*_Ω_ and the *reconstruction* of the world 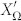. Its mutual information 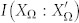 is defined as^5^,^23^,^24^: 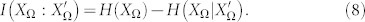
The above expression has an equivalent formulation, namely 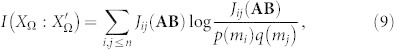
where the right side of the above equation can be identified as the *Kullback-Leibler* divergence between distributions *J*(**AB**) and *p* **·** *q*: 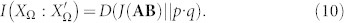
Within this formulation, the mutual information is the amount of accessory bits needed to describe the composite system *X*_Ω_, 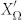 taking as the reference the distribution *p* **·** *q*, which supposes no correlation between *X*_Ω_ and 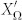.

Let us underline a feature of mutual information which is relevant for our purposes. As is well-known, max 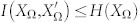, and equality holds if there is no ambiguity in the information processing process, meaning that the process is *reversible*, in logical terms. Thus, every event *m_i_* ∈ Ω has to be decoded with probability 1 to some event *m_j_* ∈ Ω which, in turn, must not be the result of the coding/decoding process of any other event. In mathematical terms, this means that **P^A^**, **Q^B^**, Λ ∈ Π*_n_*_×*n*_, being Π*_n_*_×*n*_ the set of *n* × *n* permutation matrices, which are the matrices in which every file and column contains *n* − 1 elements equal to 0 and one element equal to 1 -see Methods section. It is worth emphasizing that *δ_n_*_×*n*_, the *n* × *n* identity matrix is itself a permutation matrix. Notice that if Λ(**AB**) ≠ *δ* some symbol *m_i_* sent by the source is decoded as a different element *m_j_*. This shift has no impact on the information measure 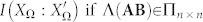, and this is one of the reasons by which it is claimed that *the content of the message* is not taken into account in the standard information measure. Actually, it is straightforward to show -see Appendix B- that only *n*! out of the (*n*!)^3^ configurations leading to the maximum mutual information also lead to a fully consistent reconstruction -i.e., a reconstruction where referential value is conserved. This mathematically shows that, for autonomous agents exchanging messages, mutual information is a weak indicator of communicative success.

### Derivation of consistent information

Now we have a complete description of the minimal system able to encompass referential values for the sent signals. It is the objective of this section to derive an information-theoretic measure, different from mutual information, that will allow us to evaluate the amount of consistently decoded information.

#### Preliminaries

The rawest evaluation of the amount of consistently decoded pairs is found by averaging the probability of having a consistent coding/decoding process during an information exchange between agent **A** and agent **B**. This corresponds to the view of an external observer simply counting events and taking into account only whether they are consistently decoded or not. This probability, denoted as *θ***_AB_**, is obtained by summing the probability of having consistent input output pair, i.e.: 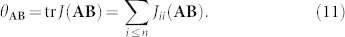
This formula has been widely used as a communicative payoff for an evolutionary dynamics in which consistent communication has a selective advantage^11^,^14^,^15^. We observe that the probability of error *p_e_*(**AB**) in this scenario is given by *p_e_*(**AB**) = 1 − *θ***_AB_**. Therefore, thanks to Fano's inequality -see Methods section-, we can relate this parameter to the information-theoretic functionals involved in the description of this problem, namely: 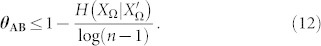
From this parameter, we can build another, a bit more elaborated functional. We are still under the viewpoint of the external observer who is now interested in the fraction of information needed to describe the composite system *X*_Ω_, 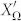 that comes from consistent input/output pairs when information is sent from **A** to **B**. This fraction, to be named *σ***_AB_**, is: 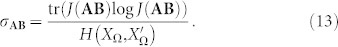
We observe that the above quantity is symmetrical in relation to *X*_Ω_ and 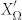. These two estimators provide global indicators of consistency of the information exchange.

#### Consistent information

However, we can go further and ask us *how much of the information from the environment is consistently decoded by agent* **B** *when receiving data from* **A**. As a first step, we observe that, since *J_ij_*(**AB**) = *p*(*m_i_*)Λ*_ij_*(**AB**), we can rewrite equation (9) as: 
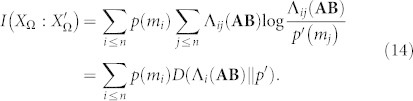
Knowing that *D*(Λ*_i_*(**AB**)||*q*) is the *information gain* associated to element *m_i_*, *p*(*m_i_*)*D*(Λ*_i_*(**AB**)||*q*) is its weighted contribution to the overall information measure. If we are interested in the amount of this information that is consistently referentiated, we have to add an “extra” weight to *p*(*m_i_*), namely Λ*_ii_*(**AB**), which is the probability of having *m_i_* both at the input of the coding process and at the output. Thus, since 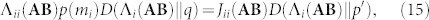
the amount of *consistent information* conveyed from agent **A** to agent **B**, 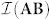, will be: 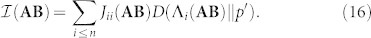
Since this is the most important equation of the text, we rewrite it using standard probability notation: 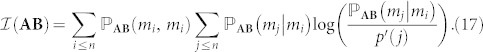
We observe that the dissipation of consistent information is due to both standard noise 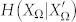, and another term, which is subtracted to 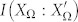, accounting for the loss of referentiality. Using equations (8, 9) and (16) we can isolate this new source of information dissipation, the *referential noise*, *ν*(**AB**), leading to: 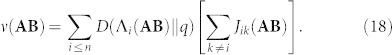
Therefore, the total loss of referential information or *total noise* will be described as 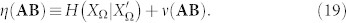
The above expression enables us to rewrite equation (16) as: 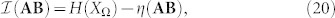
which mimics the classical Shannon Information, now with a more restrictive noise term. Interestingly, the above expression is not symmetrical: the presented formalism distinguishes the world, *X*_Ω_, from its reconstruction, 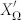. If we take into account that, according to the definition we provided for an autonomous communicating agent, the information can flow in both senses (**A** → **B** and **B** → **A**), we can compute the average success of the communicative exchange between **A** and **B**, 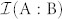, as: 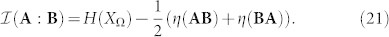

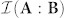
 is the *consistent information about the world* Ω *shared by agents* **A** *and* **B**. In contrast to the previous one, the above expression is now symmetrical, 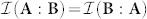, because both agents share the same world, represented by *X*_Ω_. We remark that this is an information-theoretic functional *between two communicating agents*, it is not an information-measure between two random variables, like mutual information is. This equation quantifies the communication success between two minimal communicating agents **A**, **B** transmitting messages about a shared world.

### Properties

In this section we draw several important consequences from the treatment just presented, based on the consistent information concept. The rigorous and complete proofs behind them can be found in the Methods section, together with a brief discussion about the actual consistency of this measure when applied to single agents in a population (i.e., the ‘self-consistency' or coherence that an individual agent should also keep about the world).

#### The binary symmetric channel

**Figure 3:**
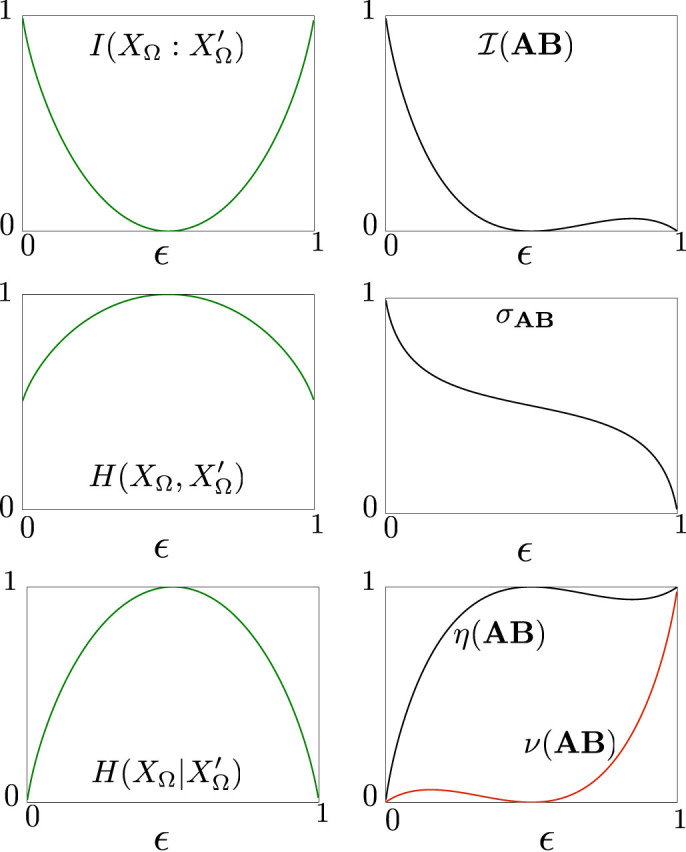
The binary symmetric channel when we enrich the communication system with a referential set shared by coder and decoder agent. Plots correspond to the different values of the binary symmetric channel along 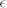, the referential shift parameter, from 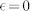 (total information with no loss of referentiality) to 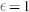 (total information with total loss of referentiality). On the left, from top to bottom, we have the classical, well known plots of 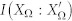, 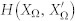 (normalized to 1) and 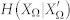. On the right, we have the equivalent ones accounting for the referentiality conservation, namely, on top, 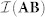, next, *σ***_AB_** and in the last plot, we have *η*(**AB**) (black line) and *ν*(**AB**) (red line). Units are given in bits. We observe that both 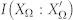 (and 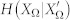) have a symmetric behavior, with a minimum (maximum) at 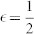 (total uncertainty). On the contrary, 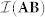 does not show a symmetric behavior, showing two minima, at 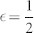 and at 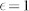. There is a local maxima at about 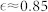, which is a by-product of the combination of the loss of uncertainty of the system and a small but non-vanishable degree of referentiality conservation.

We first consider the simplest case, from which we can easily extract analytical conclusions that help us gain intuition: the *Binary Symmetric Channel* with uniform input probabilities. We are concerned with a world Ω having two events such that *p*(1) = *p*(2) = 1/2, two agents **A** and **B** sharing information about this world, and a binary channel, Λ. The agents' and channel configuration are assumed to be of the following form: 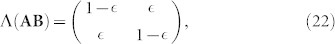
being Λ(**AB**) = **P^A^**Λ**Q^B^**, as defined at the beginning of the results section. We will refer to 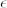 as the *referential shift*, which is the probability that a given event is wrongly decoded in the reconstruction of Ω. In this minimal system all functionals can be easily evaluated. First, we have that 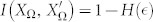, and that 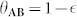, being 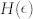 the entropy of a *Bernouilli process* having parameter 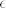 -see Methods section. This leads to the following expression of the consistent information: 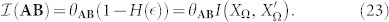
We can also easily compute *σ***_AB_**: 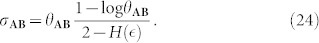
The behavior of consistently decoded information is shown in figure (3). In these plots we confronted the behavior of 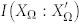, 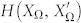 and 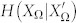 with their analogous counterparts when referentiality is taken into account, nalemy 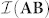 and *σ***_AB_** and *ν*(**AB**) (and *η*(**AB**)) respectively. We can observe the symmetric behavior of the first ones against 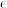, which highlights the total insensibility to referentiality conservation of these classical measures. Instead, we observe that 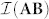, *σ***_AB_**, *η*(**AB**) and *ν*(**AB**) do reflect the loss of referentiality conservation, showing a non-symmetric behavior with a generally decreasing trend as referentiality is progressively lost.

#### Decrease of information due to referential looses

One interesting consequence of equation (23) is that, except for very restricted situations, the presence of noise has a negative impact on the value of the consistent information, leading to the general conclusion that: 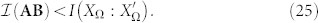
This latter inequality shows that, in most cases, in the absence of a designer, part of the information properly transmitted is actually useless for communication in a framework of autonomous agents. As demonstrated in the Methods section, the strict inequality holds in general. Indeed, the above relation becomes equality only in the very special case where there is perfect a matching between the two agents (i.e.: Λ(**AB**) = *δ_n_*_×*n*_, being *δ_n_*_×*n*_ the *n* × *n* identity matrix.) or trivially, in the case where 
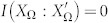
.

But we can go further. Let us consider that we know that the system displays a given value of 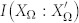 and, by assumption, we also know *H*(*X*_Ω_). In these conditions, one can easily derive 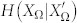 by simply computing 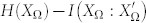. But it is possible to set a bound to the value of 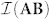 as well. As in many problems of information theory, the general case is hard, even impossible to deal with. However, several approaches become viable in special but illustrative cases. Let us assume the paradigmatic configuration in which (∀*m_i_* ∈ Ω)*p*(*m_i_*) = 1/*n* and where Λ(**AB**) acts as a symmetric channel. In this case, we have that 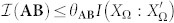, where 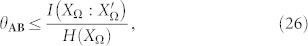
and, therefore: 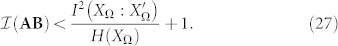
(See the Methods section for the details of the above derivations). This tells us, after some algebra, that in this framework, 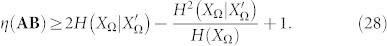
Therefore, for 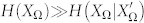, we have that 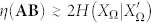, leading to 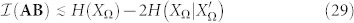
and, for example, for the case in which 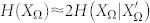 we have that: 
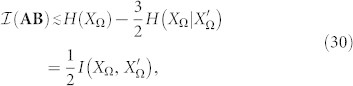
The above examples enable us to illustrate the strong impact of noise on the conservation of the referential value within a communication exchange -stronger than the one predicted by standard noise.

## Discussion

Shannon's information theory had a great, almost immediate impact in all sorts of areas, from engineering and genetics to psychology or language studies^25^. It also influenced the work of physicists, particularly those exploring the foundations of thermodynamics, who found that the entropy defined by Shannon provided powerful connections with statistical mechanics, particularly in terms of correlations. It is mainly at that level -i. e. the existence of correlations among different subsystems of a given system- that the use of information theory has proved useful. But correlations do not ensure a crucial type of coherence that seems necessary when dealing with meaningful communication: the preservation of referentiality.

In this paper we have addressed a especially relevant problem, namely the development of an information-theoretic framework able to preserve meaning. This is a first step towards a more general goal, which would involve establishing the basis for an evolutionary theory of language change including referentiality as an explicit component. We have shown that, if consistent information is considered, its value is significantly lower than mutual information in noisy scenarios. We have derived an analytical form of consistent information, which includes referential noise along with the standard noise term. Our information measure defines a non-symmetrical function and properly weights the -more strict- requirement of consistency. We have illustrated our general results by means of the analysis of a classical, minimal scenario defined by the binary symmetric channel. The approach taken here should be considered as the formally appropriate framework to study the evolution of communication among embodied agents, where the presence of consistency is inevitable due to shared perception constraints. Moreover, it might also be useful as a consistent mathematical framework to deal with cognitive-based models of brain-language evolution^26^,^27^,^28^. At this point, we should point out an important issue: Consistency of the communicative exchange is here evaluated between agents, not internally to a given agent *talking to itself*. Actually, there is no a priori any correlation between the coding and the decoding modules of a given agent. In doing so, we take the viewpoint proposed by [14] and [15]. Other approaches assumed an explicit link between the coding and decoding modules of the agent, thereby avoiding from the beginning the paradoxical situation in which two agents perfectly understand each other but, at the same time, they are not able to understand themselves [10, 11]. However, as shown in^29^, this situation is unlikely to occur under selective pressures, for the frameworks depicted by these earlier works. In the Methods section is shown that the proposed framework has also the same property, i.e., that the maximisation of consistent communication in a given community of agents leads to the self-consistency of each of them, without the need of imposing it externally, thereby simplifying the mathematical apparatus.

The framework we have developed is somehow inspired by Saussure's duality of sign: a (linguistic) sign is a twofold entity compounded of a signifier and a signified. However, it must be mentioned that there is a substantial difference between the theory we have developed and a Saussurean approach. According to Saussure, the relation between a signifier and a signified is fixed with respect to the linguistic community that uses the sign. “The masses have no voice in the matter, and the signifier chosen by language could be replaced by no other”. Saussure adopts therefore a ‘static' approach to the study of signs, whereas we adopt a dynamic perspective that allows us to address the possibility that different agents assign different meanings to the same symbol, in which case referentiality is not preserved. In this way we extend evolutionary game-theoretic arguments in order to derive a measure of consistency of the shared information between agents by incorporating the (non-)preservation of referentiality.

In the presented work we took the simplest possible form of meaning, namely, its referential object. However, we said nothing about the object itself. Further works might explore the inclusion in the above proposed framework an explicit quantification of meaning beyond its referential value, to rank events of the world and to refine the role of the information functional to evaluate proper communication exchanges in selective scenarios. In addition, new hallmarks beyond the agent-channel-agent should be explored, leading to new forms of information which play a role in biological organisation and which are poorly reflected in such a schema.

## Methods

### Definitions

#### Information theoretic functionals

The following definitions are intended to be minimal. We refer the interested reader to any standard textbook on information theory, such as [23] or [24].

- Given a random variable *X*_Ω_ taking values over the set Ω following a probability distribution *p*, 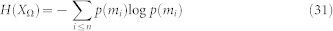
is the standard *Shannon* or *statistical* entropy.
- Given two random variables, *X*_Ω_ and 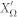, 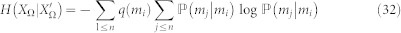
is the *conditional* entropy of *X*_Ω_ with respect 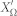, being, in that case, 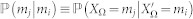. Additionally, 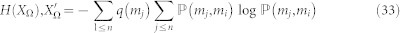
where 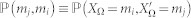 is the *joint entropy* of the two random variables *X*_Ω_, 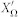.
- Given two probability distributions *π*_1_, *π*_2_ defined over the set Ω, the *Kullback-Leibler* divergence of *relative entropy* of *π*_1_ with respect *π*_2_ is: 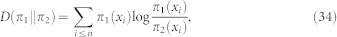
which is the amount of extra information we need to describe *π*_1_ taking as the reference distribution *π*_2_.
- *Fano*'s inequality. The probability of error in decoding is bounded satisfies the following inequality: 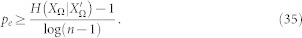
- A Bernoulli process is a stochastic process described by a random variable *X* taking value in the set *A* = {0, 1}, being 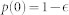 and 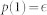. 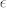 is the *parameter* of the Bernoulli process. Its entropy *H*(*X*) is commonly referred as 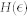, since it only depends on this parameter: 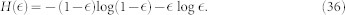

#### Permutation matrices

A *permutation matrix* is a square matrix which has exactly one entry equal to 1 in each row and each column and 0's elsewhere. For example, if *n* = 3, we have 6 permutation matrices, namely: 
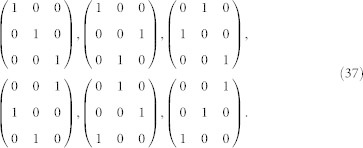
The set of *n* × *n* permutation matrices is indicated as Π*_n_*_×*n*_ and it can be shown that, if **A** ∈ Π*_n_*_×*n*_, **A**^−1^ = **A***^T^* ∈ Π*_n_*_×*n*_ and, if **A**, **B** ∈ Π*_n_*_×*n*_, the product **AB** ∈ Π*_n_*_×*n*_. Furthermore, it is clear that *δ_n_*_×*n*_ ∈ Π*_n_*_×*n*_, being *δ* the identity matrix or *Kronecker* symbol, defined as *δ_ij_* = 1 if *i* = *j* and *δ_ij_* = 0, otherwise.

### Inequalities

We present the inequalities described in the main text in terms of three lemmas on the upper bounds of 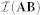. The first one concerns inequality (25). The second one is general and supports the third, which proves inequality (27):

*Lemma 1*.- Let **AB** be two agents sharing the world Ω. The Amount of *consistent* information transmitted from **A** to **B** -when **A** acts as the coder agent and **B** as the decoder one- satisfies that 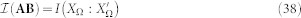
only in the following two extreme cases:

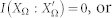
, or
2. Λ(**AB**) = *δ_n_*_×*n*_.

Otherwise, 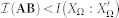.

*Proof*.- The first case is the trivial one in which there is no information available due to total uncertainty -corresponding to 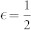 in the case of the symmetric binary channel studied above, see also figure (3). The second one is more interesting. Indeed, having Λ(**AB**) = *δ* means that 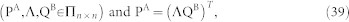
where we use that, if **C** ∈ Π*_n_*_×*n*_, **C**^−1^ = **C***^T^*, also having that **C***^T^* ∈ Π*_n_*_×*n*_. Out of these two situations, ∃*J_ik_*(**AB**) > 0, in which *i* ≠ *k*, since there are more than *n* non-zero entries in the matrix Λ(**AB**), leading to 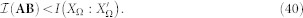


*Lemma 2*.- Let **AB** be two agents sharing the world Ω. The Amount of *consistent* information transmitted from **A** to **B** -when **A** acts as the coder agent and **B** as the decoder one- is bounded as follows: 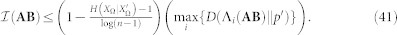


*Proof*.- Let 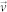 and 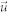 be two vectors of 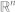. Its scalar product, 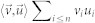, is bounded, thanks to the so-called *Hölder's inequality*, in the following way: 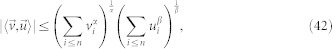
as long as *α* and *β* are *Hölder conjugates*, i.e., 1/*α* + 1/*β* = 1. The above expression can be rewritten, using the notation of norms as 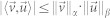 -recall that, for *α* = *β* = 1/2 we recover the well-known *Schwartz inequality* for the euclidean distance. If we put *α* → 1 and *β* → ∞ we obtain 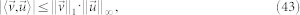
where 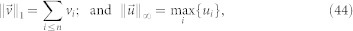
being the last one the so-called *Chebyshev's norm*. Now we want to apply this machinery to our problem. The key point is to realize that 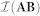 can be expressed as a scalar product between two vectors, having the first one coordinates *J*_11_(**AB**), …, *J_nn_*(**AB**) and the second one *D*(Λ_1_(**AB**)||*q*), …, *D*(Λ*_n_*(**AB**)||*p*′). We remark that this step is legitimated because all the terms involved in the computation are positive. Therefore, by applying the Hölder's inequality over the definition of 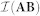, we have that 
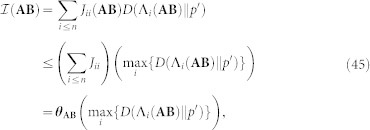
being *θ***_AB_** defined in equation (11). Now we observe that the probability of error in referentiating a given event of 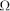 is *p_e_* = 1 − *θ***_AB_**. This enables us to use Fano's inequality to bound *θ***_AB_**: 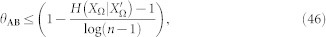
thereby obtaining the desired result.

*Lemma 3*.- (Derivation of inequality (27)). Let **AB** be two agents sharing the world 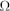 and such that (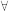
*m_i_* ∈ 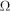)*p*(*m_i_*) = 1/*n* and that the channel defined by Λ(**AB**) is symmetric. Then, the following inequality holds: 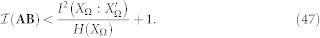


*Proof*.- The first issue is to show that, if (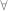
*m_i_* ∈ Ω)*p*(*m_i_*) = 1/*n* and the channel defined by Λ(**AB**) is symmetric, then 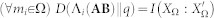. Indeed, since the channel is symmetric *p* = *p*′ and thus 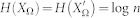. Then take any *m_i_* ∈ Ω and compute *D*(Λ*_i_*(**AB**)||*p*′): 
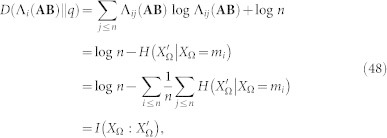
where in the third step we used the property that, in a symmetric channel, (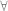
*m_i_*, *m_j_* ∈ Ω) 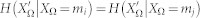. Thus, if we average a constant value, we obtain such a value as the outcome (last step). Then, we apply inequality (41): 
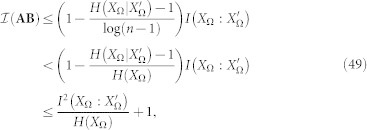
where, in the second step we used the fact that *H*(*X*_Ω_) = log *n* > log(*n* − 1) and in the third step we bound the remaining term 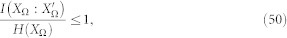
since 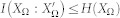, thus completing the proof.

### Achieving self-consistency maximizing consistent information

The structure of the functional accounting for the amount of consistent information shared by two agents -equation (21)- can lead to the paradoxical situation in which high scores on 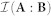 do not imply high values of 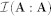 or 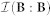. In brief, the degeneracy of possible optimal configurations seems to jeopardize self-understanding even in the case in which communication is optimal. Interestingly, this apparent paradox can be ruled out if we consider a population of agents, for several representative cases, as demonstrated in^29^ using a version of *θ***_AB_**. For the particular case where *η_AB_* = 0, we have seen at the beginning of this section that 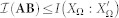, having the equality only in the special case in which Λ(**AB**) = *δ_n_*_×*n*_, which, in turn, implies that 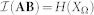. The interesting issue is that in the presence of three or more agents **A**, **B** and **C**: 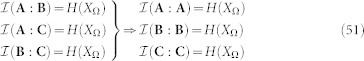
i.e., maximizing the communicative success over a population of agents results automatically in a population of self-consistent agents, although there is no a-priori correlation between the coder and the decoder module of a given agent. Now we rigorously demonstrate this statement.

*Lemma 3*.- Let us have three **A***_i_*, **A***_j_*, **A***_k_* agents communicatively interacting and sharing the world 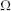. Then, if 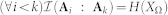, then 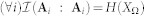.

*Proof*.- We observe, as discussed above, that the premise only holds if (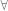
*i* < *k*) 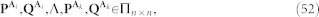
and 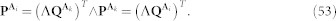
Now we observe that, if 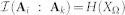, 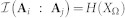, we conclude that: 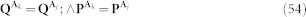
i.e., **A***_k_* = **A***_j_*. Now, knowing that 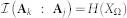, then: 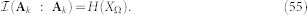
We can easily generalize this reasoning to an arbitrarily large number of communicating agents.

## Author Contributions

B.C.-M., J.F. and R.S. designed research, made analytic derivations and wrote the paper.

## Figures and Tables

**Figure 1:**
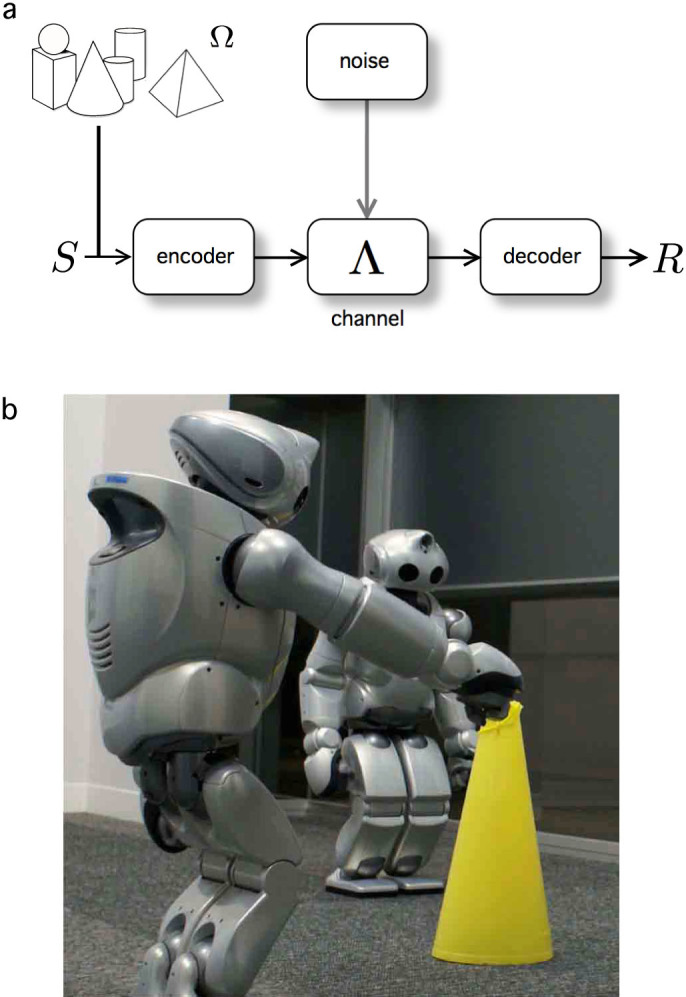
In standard theory of information, as defined in Shannon's theory, a communication system (a) is described in terms of a sequential chain of steps connecting a source of messages (S) and a final receiver (R). The source can be considered linked to some external repertoire of objects (Ω). An encoder and a decoder participate in the process and are tied through a channel Λ, subject to noise. The acquisition and evolution of a language, as it happens in artificial systems of interacting agents, like robots (b), involves some additional aspects that are usually ignored in the original formulation of Shannon's approach. Those include the embodiment of agents and the necessary consistency in their communicative exchanges emerging from the their perceptions of the shared, external world. Picture courtesy of Luc Steels.
